# Knowledge attitude and practice regarding diabetes mellitus among Nondiabetic and diabetic study participants in Bangladesh

**DOI:** 10.1186/s12889-017-4285-9

**Published:** 2017-04-26

**Authors:** Kaniz Fatema, Sharmin Hossain, Khurshid Natasha, Hasina Akhter Chowdhury, Jesmin Akter, Tahmina Khan, Liaquat Ali

**Affiliations:** 1 Department of Epidemiology, Bangladesh University of Health Sciences (BUHS), 125/1 Darus Salam, Mirpur, Dhaka, 1216 Bangladesh; 2 Department of Health Promotion and Health Education, BUHS, 125/1 Darus Salam, Mirpur, Dhaka, 1216 Bangladesh; 3 Department of Biostatistics, BUHS, 125/1 Darus Salam, Mirpur, Dhaka, 1216 Bangladesh; 4 Department of Reproductive and Child Health, BUHS, 125/1 Darus Salam, Mirpur, Dhaka, 1216 Bangladesh; 5 Department of Biochemistry and Cell Biology, BUHS, 125/1 Darus Salam, Mirpur, Dhaka, 1216 Bangladesh; 6 Present address: Faculty of Medicine, University of New South Wales (UNSW), Sydney, NSW 2052 Australia

**Keywords:** Knowledge, Attitude, Practice, Type 2 diabetes, Bangladesh

## Abstract

**Background:**

Increased awareness amongst large population groups is a major determinant for the prevention of diabetes and its complications as well as related metabolic disorders. Knowledge and attitude are the principal markers of awareness that need to be studied in various population groups in specific racial and cultural contexts. The present study was undertaken to explore knowledge, attitude and practice (KAP) regarding -diabetes mellitus (DM) among nondiabetic (nonDM) and type 2 diabetes mellitus (T2DM) patients in Bangladesh.

**Methods:**

A cross-sectional study was conducted among 18,697 adults (aged 18 years and above; 7796 male and 10,901 female; 6780 nonDM and 11,917 T2DM) selected purposively from the OPD of 19 healthcare centres in and around Dhaka and in northern parts of Bangladesh. KAP were assessed by a pre-structured, interviewer-administered questionnaire and categorised using predefined scores of poor (<mean - 1 SD), average (mean ± 1 SD) and good (>mean + 1 SD). Univariate and bivariate statistical analysis were done as appropriate. Multivariate linear regression was done to examine the association between diabetes related KAP and other covariates.

**Results:**

The mean (±SD) age (years) of all the study participants was 46 ± 14, mean BMI 24.4 ± 4.1 and mean waist-hip ratio (WHR) was 0.93 ± 0.07. The proportion of poor, average and good knowledge scores among T2DM subjects were 17%, 68% and 15% respectively. The corresponding values for attitude score were 23%, 67% and 10% respectively. The KAP regarding diabetes was found to be better among people who were living with diabetes compared to their counterparts. DM males showed better knowledge and practice regarding diabetes, compared to nonDM counterparts (M ± SD; 44.18 ± 16.13 vs 40.88 ± 15.62, p = <0.001; 66.00 ± 29.68 vs 64.21 ± 31.79, *p* < 0.001, respectively). Females showed better attitude score compared to males. Overall KAP were found to be significantly higher (*p* < 0.001) in middle aged (31–50 years) participants in each group. Participants from urban residents, higher educational background and upper socio-economic class demonstrated significantly greater score in terms of KAP in both nonDM and T2DM groups (*p* < 0.001). On linear regression analysis, knowledge scores correlated strongly with education, income, residence, diabetic state, BMI and attitude.

**Conclusions:**

The overall level of knowledge and practice concerning diabetes among Bangladeshi population is average, but the overall level of attitude is good both in nonDM and T2DM subjects. To prevent diabetes and its complications there is an urgent need for coordinated educational campaigns with a prioritized focus on poorer, rural and less educated groups.

## Background

Diabetes mellitus (DM) is one of the major fast growing noncommunicable disease (NCD) threats to global public health. Trends in the incidence of diabetes indicate a disproportionate increase in developing countries due to current rapid demographic transitions from traditional to more westernized and urbanized lifestyles [1].

A United Nations (UN) resolution in 2007 confirmed diabetes mellitus as a significant global public health issue [2]. A decade ago, diabetes was not considered as a major public health problem in developing countries like Bangladesh, whereas by 2015 the situation has changed dramatically. In 2012, the International Diabetes Federation (IDF) estimated that 3.8 million or 4.8% of people living in Bangladesh have diabetes. By 2025 the number is expected to grow to 7.4 million or 6.1% of the population. In terms of the number of people living with diabetes, this explosion will shift Bangladesh from tenth to seventh among the top ten countries by the year 2025. By then, 80% of all diabetes cases will occur in the low and middle socioeconomic classes where knowledge about diabetes is poor [3].

Knowledge plays a vital role in any future disease development and its early prevention and detection. Positive knowledge, attitude and practice (KAP) are important for DM patients. Elements of KAP are interrelated and dependent on each other. If the level of one element is higher, the other two factors should be affected positively. KAP regarding diabetes vary greatly depending on socio economic conditions, cultural beliefs and habits [4].

Knowledge of diabetes can prevent the imminent chronic comorbidities of DM, which impact significantly on the quality of life of the diabetic patients. Information can help people to assess their risk of diabetes, motivate them to seek proper treatment and care, and inspire them to take charge of their disease for their lifetime [5]. A literature search on knowledge about diabetes in developing countries yielded very few studies dealing with awareness of diabetes among people with the disease [6, 7] and there are virtually no data on whole populations. In Bangladesh there have been a few clinical based studies on knowledge about diabetes among nondiabetic and diabetic patients [8, 9]. Even in other developing countries, such studies have focused mainly on diabetic patients and are mostly clinic based. The necessity for greater awareness regarding prevention, diagnosis, risk factor control and disease management has been supported from previous KAP studies [10–15]. There is a need to investigate KAP levels among participants living with diabetes to aid in future development of programs and techniques for effective health education. This study was designed to investigate diabetes related knowledge, attitude and practices of non-diabetic (nonDM) and type 2 diabetes mellitus (T2DM) populations in rural, semi-urban and urban areas of Bangladesh.

## Methods

### Subjects and methods

**Fig. 1:**
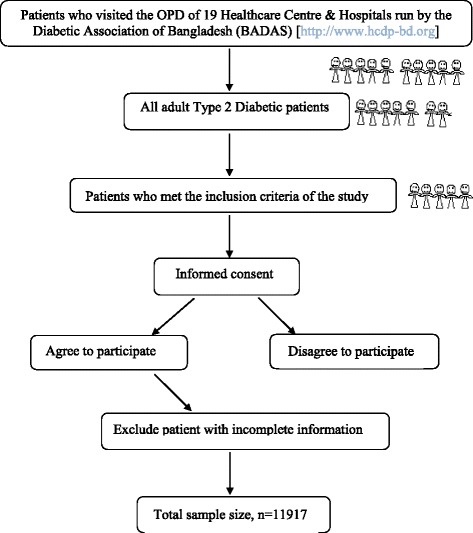
Flow chart for the selection of subjects

The survey was conducted from October 2010 to March 2011. It was a cross-sectional study conducted among 18,697 adult subjects (aged 18 years and above; 7796 males and 10,901 females, 6780 nonDM and 11,917 T2DM) who were attending the outpatient departments (OPDs) of total nineteen (19) health care centres and hospitals run by the Diabetic Association of Bangladesh (BADAS) in and around the capital city Dhaka and in the northern parts of Bangladesh [*Details available at*: http://www.hcdp-bd.org]. Areas defined as rural in this study still represent the truly countryside characteristics of Bangladesh but due to rapid urbanization they might be classified as semiurban in near future. The rationale for choosing the areas is to observe the transition of the disease as a consequence of changing lifestyles. These areas were included as 75% of the total population of Bangladesh live in such areas [16]. Using the purposive sampling method all subjects attending the health care facilities under the study who fulfilled the inclusion criteria and showed willingness to participate were included in this study (Flow chart in Fig. 1).

**Fig. 2:**
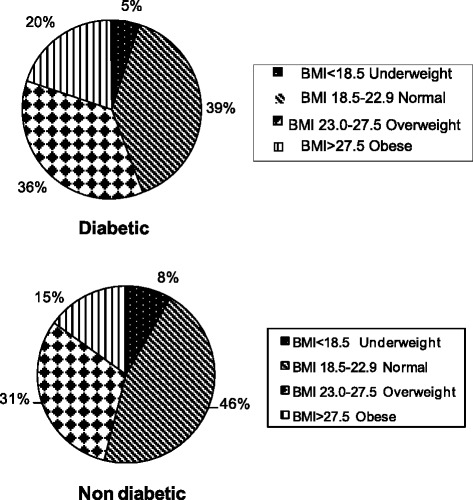
Distribution of the study subjects according to Body Mass Index (BMI)

**Fig. 3:**
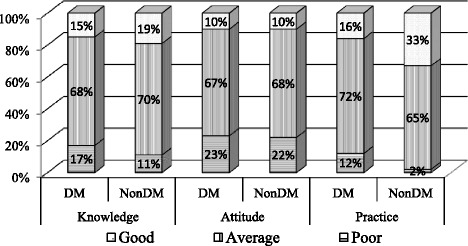
Levels of knowledge, attitude and practice regarding diabetes among the study subjects

Patients who had severe physical and mental illness were excluded. Opinion and advice were obtained from teachers, experts from relevant fields, and advisors throughout the initial period of constructing the questionnaire. The questionnaire were constructed in their native language of Bangla and were kept as simple as possible where a pre-arranged sequence was also maintained. Pretesting of the questionnaire was performed to gather information on its understandability, time consumed by each question, consistency among related variables and acceptability. After pretesting, a pre-programmed, interviewer administered questionnaire, based on Oracle DBMS software, was used for final data collection.

The software was divided into two sections according to the questionnaire - Section 1 consisted of socio-demographic information, family history of the disease, anthropometric measurements (i.e.*; height, weight, waist and hip circumferences*), and clinical findings (*systolic and diastolic blood pressure measurement*) and biochemical parameters (*glycemic and lipid profile* i.e.*, total cholesterol, triglyceride, high density lipoprotein and low density lipoprotein*) reports. Section 2 consisted of issues related to knowledge, attitude and self-care practice and patients’ lifestyle (*food intake pattern, physical activity, smoking* etc.).

Data were collected individually by twenty (20) data collectors. For standardized data collection all the collectors were given extensive training over one week on relevant issues like patient counselling, nutritional information, anthropometric measurement, and cross checking of answers. Anthropometric measurements (height, weight, waist and hip) were done using standard techniques. BMI and WHR were calculated using appropriate formula. A BMI of 23 was taken as the cut-off point for the overweight category, as defined by the World Health Organization (WHO) for this population [17]. Biochemical reports of the patients were collected from the patient record book.

#### KAP questionnaire

The questions relevant to KAP in the questionnaire were derived from the validated instruments: (i) Knowledge and Awareness of Diabetes Questionnaire developed for the Chennai Urban Rural Epidemiology Study [18], (ii) AusDiab Health Knowledge, Attitudes and Practices Questionnaire 99/00 [19], and (iii) KAP construction guides [20].

Questions evaluating knowledge of diabetes were follows:

1. What is diabetes?
2. Do you know diabetes is a genetic/hereditary disease?
3. What is dyslipidemia/obesity/hypertension?
4. What are the risk factors of diabetes/dyslipidemia/obesity/hypertension?
5. Do you know how to measure diabetes/dyslipidemia/obesity/hypertension?
6. Do you know the complications due to diabetes/dyslipidemia/obesity/hypertension?
7. Do you know the effect of regular exercise on diabetes?
8. Do you know the effect of healthy dietary habit (timing and food intake pattern, extra salt intake) on diabetes?
9. Do you know the effect of sugar on diabetes?
10. Do you know how active/passive smoking affects diabetes?

Questions evaluating practice of diabetes control and management particularly among those who have been diagnosed with diabetes were as follows:

1. How often (daily/weekly/monthly) do you visit to physicians, monitor blood glucose and blood pressure?
2. Do you control your weight?
3. Do you take food timely?
4. Do you add extra salt to your regular diet?
5. How much time do you spend for exercise?
6. Do you smoke?
7. Do you have any exposure to passive smoking?

#### KAP scoring

For analysis, a total of 16 items were included in the knowledge section which included elementary knowledge of diabetes, benefits of exercise, complications of diabetes, groupings of foods and their exchange list, ideal body weight and obesity. For the sixteen items knowledge question, the maximum attainable score was ‘16’ and the minimum score was ‘0’.

Likewise, in the attitude section, a total of 8 items were included which consisted of respondents attitude towards diabetes. A three point Likert scale was used to measure attitude. Questions evaluating attitude towards the treatment of diabetes were associated with the three categories of response: ‘agree’, ‘neither agree nor disagree’ and ‘disagree’. Each positive response (agree) was assigned a score of ‘1’, and each negative response as a score of ‘0’. For the eight attitude related questions the maximum attainable score was ‘8’ and the minimum score was ‘0’. In the same manner, for the eight items in the practice category, such as glucose monitoring, physician visit, weight management, exercise, foot care, smoking, consumption of betel nuts, extra salt intake, groupings of foods and their exchange list, the maximum attainable score was ‘8’ and minimum was ‘0’. The combined level of knowledge, attitude and practice (KAP) was classified according to each respondent’s score. Poor knowledge and practice corresponded to a score of (<Mean – 1 SD); average knowledge and practice corresponded to a score between (Mean ± 1 SD); good knowledge and practice corresponded to a score of (>Mean ± 1 SD) [21]. Informed written consent was obtained from all respondents after a full explanation of the nature, purpose, and procedures used for the study. Participants were informed about their right to withdraw from the study at any stage of the study. Ethical approval was obtained from the ethics and Research Review Committee of the Diabetic Association of Bangladesh.

### Operational definition

**Knowledge:** ‘Knowledge in this study was defined as the understanding of information regarding diabetes on 16 items’.

**Attitude:** ‘Attitude in this study was defined as the approach of the populations towards the 9 items related to diabetes’.

**Practice:** ‘Practice in this study was defined as the pattern and regularity of practices of the 10 items related to diabetes’.

### Data editing and statistical analysis

Data editing was carried out by checking and verifying the completed questionnaire at the end of the interview, as well as at the end of whole survey and before the analysis. The data analysis was performed using Statistical Package for Social Science (SPSS) version 16.0.

Respondent’s socio-demographic characteristics were stated using descriptive statistics. Means, standard deviations, and proportions were generated to describe the overall sample characteristics (age, gender, living area, monthly income, and education), diabetic status and BMI. t-test and ANOVA were used to test the equality of means between nonDM and DM groups.

Multivariable linear regression modelling was applied to determine the variable associated with diabetes related KAP. All association were considered significant at the alpha level of 0.05.

## Results

For the total 18,697 study participants, the mean (±SD) age was 46 ± 14 years. Among them, a female (*n* = 10,901, 58%) preponderance was observed. A higher proportion of the subject (53%) lived in rural area. Most of them (36%) had primary education (which means upto 8th grade of school from June 2016), while 32% of them had upto higher secondary education. Next were those with graduate level education (19%) and those who never attended school (13%). Professionally, 28% were homemakers, 25% service holders, 33% were business persons and 14% were others service providers. Slightly more than half of the respondents belonged to the lower-middle-income group, one-fifth belonged to the low-income group, approximately 21% belonged to the upper-middle income group, and only 4% belonged to the high-income group (Table 1).

The mean (±SD) BMI of the total study subjects was 24.1 ± 4.07. Underweight and normal BMIs were found more frequently in the nonDM subjects compared to the T2DM subjects. However, around 40% of both groups of participants had BMI within the normal range (18.5–23.0) (Fig. 2).

The mean (±SD) knowledge, attitude and practice score of the respondents were 41 ± 16, 85 ± 12 and 57 ± 30, respectively. Among the nonDM, the levels of knowledge were poor in 11%, moderate in 70% and good in 19% of the subjects. The levels of attitude were also described accordingly as poor 22%, moderate 68% and good 10%. The levels of practice of the study subjects were found to be poor in 2%, moderate in 65% and good in 33% cases. However for diabetic participants, the levels of knowledge were poor in 17%, moderate in 68% and good in 15% subjects. The levels of attitude were also described accordingly as poor 23%, moderate 67% and good 10%. The levels of practice of study participants were found to be poor in 12%, moderate in 72% and good in 16% of the subjects (Fig. 3).

The KAP towards diabetes was found to be better among people who were living with diabetes compared to people without diabetes. DM males scored both in knowledge and practice, compared to their counterparts (M ± SD; male vs female; 44.18 ± 16.13 vs 40.88 ± 15.62, *p* < 0.001; 66.00 ± 29.68 vs 64.21 ± 31.79, *p* < 0.001, respectively). However, females showed better attitude compared to males. Overall KAP were found to be significantly higher (*p* < 0.001) in middle aged (31–50 years) participants in each group. In general, participants from urban residence and upper socioeconomic class with higher educational background, demonstrated significantly greater scores in terms of KAP both in nonDM and DM groups (*p* < 0.001) (Table 2).

The one way analysis of variance (ANOVA) showed significant differences between the mean scores for knowledge, attitude and practice in the various categories for all the covariates, namely area, income, education and BMI except for the attitude score for BMI. The level of education positively correlated with knowledge and attitude scores (*p* < 0.05). The pattern was random for income and BMI.

Multiple linear regressions for the total knowledge scores, total practice scores, and total attitudes scores on covariates identified in the bi-variates analysis showed several significant (adjusted) associations. Table 3 shows the results for the KAP score. Regression analysis showed that the knowledge score is associated with education, income, residential area, diabetic state, BMI and attitude when knowledge was put as a dependent variable and the covariates of age, sex, area, level of education, diabetic state, and body mass index as independent variables. On the other hand, the attitude score was found to be associated with age, sex, education, income, residence and knowledge when attitudes was put as dependent and other confounding variables (diabetic state and BMI) were adjusted. The same formula was used for ‘practice’ also (Table 3).

**Table 1:** Characteristics of the study subjects (*N* = 18,697)

Variables	Number	Percentage (%)
Age (mean ± SD), years	46 ± 14	-
≤30 yrs	2900	15.5
31 to 50 yrs	8848	47.3
≥51 yrs	6949	37.2
Gender		
Male	7796	42
Female	10,901	58
Living area		
Urban	8732	47
Rural	9965	53
Education		
Illiterate	2433	13
Primary to 8th grade	6692	36
Secondary to higher secondary	6011	32
Graduate & above	3561	19
Monthly Income (US$)		
Low income (≤905)	3461	19
Lower-middle income (906–3595)	10,708	57
Upper-middle income (3596–11,115)	3834	21
High income (≥11,116)	694	4
Occupation		
Service	4674	25
Business	6170	33
Homemaker	5235	28
Others (laborer/ unemployed)	2618	14

Results are expressed as number (%) and mean ± SD; 1 US$ = 80 Bangladeshi Taka (BDT)

Primary means upto Class VIII in Bangladesh from June 2016

**Table 2:** Knowledge, attitude and practice score of the study subjects according to different variables (*N* = 18,697)

Variables			NDM group (*n* = 6780)		DM group (*n* = 11,917)		
		Knowledge Score (%)	Attitude Score (%)	Practice Score (%)	Knowledge Score (%)	Attitude Score (%)	Practice Score (%)
Sex	Male	40.61 ± 15.04	89.99 ± 15.91	42.56 ± 24.04	44.18 ± 16.13	89.87 ± 15.01	65.99 ± 29.68
	Female	38.65 ± 15.11	91.25 ± 12.95	43.62 ± 25.13	40.88 ± 15.62	90.41 ± 14.35	64.21 ± 31.79
	*t/p value* ^a^	5.362/0.001	−3.575/0.001	−1.766/0.078	11.004/0.001	−1.960/0.50	3.036/0.002
Age (years)	18 to 30	40.22 ± 14.74	91.01 ± 13.69	41.93 ± 23.65	39.34 ± 14.74	89.71 ± 15.64	62.97 ± 30.70
	31 to 50	39.80 ± 15.22	90.64 ± 15.46	42.76 ± 24.87	42.80 ± 15.18	90.75 ± 13.68	64.89 ± 31.16
	51 & above	38.25 ± 15.38	89.94 ± 14.51	45.80 ± 25.30	41.70 ± 16.65	89.72 ± 15.34	65.06 ± 30.94
	*F/p value* ^a^	7.579/0.001	2.322/0.098	11.107/0.001	16.859/0.001	7.402/0.001	1.299/0.273
Area	Urban	43.15 ± 16.01	90.62 ± 14.24	46.53 ± 25.14	44.38 ± 17.02	89.58 ± 16.09	64.72 ± 30.99
	Rural	36.75 ± 13.68	90.63 ± 14.73	40.29 ± 23.77	40.02 ± 14.47	90.78 ± 13.06	65.01 ± 31.08
	*t/p value* ^a^	17.766/0.001	−0.032/0.974	10.472/0.001	15.084/0.001	−4.491/0.001	−0.515/0.001
Education	Illiterate	26.80 ± 10.25	86.90 ± 19.41	38.49 ± 22.37	31.03 ± 12.20	88.90 ± 15.42	60.62 ± 28.64
	Primary to 8th grade	34.23 ± 12.45	90.46 ± 14.49	39.721 ± 23.32	39.06 ± 14.45	90.08 ± 15.48	63.98 ± 30.57
	Secondary to higher secondary	40.41 ± 13.27	92.09 ± 12.92	42.38 ± 24.38	45.46 ± 14.76	91.44 ± 12.80	65.13 ± 33.07
	Graduate & above	46.77 ± 15.04	91.01 ± 13.57	47.25 ± 25.56	50.08 ± 15.74	90.17 ± 14.02	68.31 ± 30.99
	*F/p value* ^a^	575.073/0.001	20.893/0.001	48.094/0.001	755.416/0.001	10.548/0.001	25.109/0.001
Monthly Family Income (in US$)	Low income (≤905)	32.17 ± 12.40	90.38 ± 15.78	37.50 ± 22.91	35.40 ± 14.01	90.61 ± 14.71	62.83 ± 30.52
	Lower-middle income (906–3595)	40.47 ± 14.63	90.86 ± 13.79	44.02 ± 24.50	42.57 ± 15.39	90.52 ± 14.07	65.66 ± 30.89
	Upper-middle income (3596–11,115)	45.75 ± 15.50	90.47 ± 14.84	47.58 ± 25.26	46.82 ± 16.29	89.28 ± 15.47	64.97 ± 31.66
	High income (≥11,116)	47.71 ± 15.11	89.55 ± 15.53	44.70 ± 27.15	48.85 ± 17.25	88.59 ± 15.88	65.09 ± 32.22
	*F/p value* ^a^	258.954/0.001	0.882/0.450	46.729/0.001	275.329/0.001	6.759/0.001	5.202/0.001
BMI	Underweight	33.59 ± 13.44	90.31 ± 15.52	35.57 ± 22.28	33.16 ± 14.09	90.26 ± 14.66	61.24 ± 28.76
	Normal	38.20 ± 14.83±	90.46 ± 14.90	41.64 ± 24.43	40.96 ± 15.68	90.15 ± 14.61	65.08 ± 30.86
	Overweight	42.14 ± 15.04	90.85 ± 13.51	45.6 ± 24.85	43.68 ± 15.82	90.54 ± 13.98	66.26 ± 30.66
	Obese	42.10 ± 15.49	90.83 ± 14.72	46.46 ± 24.55	43.88 ± 15.88	89.70 ± 15.61	62.90 ± 32.46
	*F/p value* ^a^	69.586/0.001	0.450/0.717	35.263/0.001	101.537/0.001	1.717/0.161	9.022/0.001

Results are expressed as number (%) and mean ± SD; NDM, non-diabetes mellitus; DM, Diabetes mellitus; BMI, body mass index^; a^For categorical variables *p*-values were obtained by doing independent samples t-test and or ANOVA where appropriate.

**Table 3:** Association of socio-demographic characteristics with Knowledge, Attitude and Practice

a. Dependent variable: Knowledge**				
	b ^1*^	Standard error	Beta ^2*^	*p*
(Constant)	24.496	1.267		0.000
Sex	0.348	0.235	0.011	0.140
Area	−1.805	0.181	−0.068	0.000
Age	−0.013	0.008	−0.011	0.126
Education	4.301	0.079	0.400	0.000
Income	1.111	0.000	0.013	0.040
Diabetic State	−3.982	0.235	−0.122	0.000
BMI	0.400	0.027	0.100	0.000
Attitude	0.076	0.007	0.071	0.000
b. Dependent Variable: Attitude**				
(Constant)	88.418	1.155		0.000
Sex	1.080	0.243	0.037	0.000
Area	−0.107	0.187	−0.004	0.568
Age	−0.027	0.009	−0.026	0.002
Education	0.044	0.088	0.004	0.618
Income	−3.634	0.000	−0.005	0.517
Diabetic state	0.361	0.245	0.012	0.141
BMI	-0.094	0.028	−0.025	0.001
Knowledge	0.082	0.008	0.088	0.000
c. Dependent Variable: Practice**				
(Constant)	58.627	2.545		0.000
Sex	1.504	0.477	0.024	0.002
Area	0.293	0.368	0.006	0.425
Age	0.946	0.159	0.045	0.000
Education	1.208	0.173	0.057	0.000
income	2.887	0.000	0.002	0.793
Diabetic State	−20.509	0.479	−0.321	0.000
BMI	-0.022	0.056	−0.003	0.689
Knowledge	0.235	0.015	0.120	0.000
Attitude	0.088	0.014	0.042	0.000

*[1 = Unstandardized sample regression co- efficient; 2 = Standardized sample regression co- efficient];

** Adjusted Ra^2^ (a) for Knowledge - 19.9%, (b) for Attitude −0.00081%, (c) for Practice - 0.2%

## Discussion

Knowledge, attitude and practice (KAP) regarding diabetes vary greatly depending on socioeconomic conditions, cultural beliefs and habits. Understanding these variables is important in designing prevention and management strategies for diabetes. The findings of the present study reassert the gaps in knowledge, attitude and practice regarding DM. Control of obesity is important for better glycemic control and prevention of complications, but it is evident from the present studies that DM subjects do not attain this ideal goal as more than half of them are overweight and obese. Obesity has been shown to be a major risk factor for T2DM. This is in line with a previous study conducted in Malaysia and also with several other studies elsewhere [22–27]. Since it is not easy for everybody to understand the concept of BMI it has been suggested to use WHR as a crude parameter for its easier understandability [28]. Another factor of concern was raised in a Pakistani study which revealed that a majority of the patients have a wrong perception to assess their weight and most overweighed patients do not consider themselves to be in that group [7].

Few studies are available which explore the relationship between knowledge and practice among nondiabetic and T2DM groups. It has been reported that people living with DM have better KAP scores towards diabetes compared to nonDM subjects [6, 21, 29–32]. In the present study the participants’ knowledge was assessed based on their understanding of DM, which included the causes, risk factors, symptoms, complications and treatment options. The diabetes related knowledge level was found to be average among nonDM (70%) and T2DM (68%) respondents. This finding is in line with two other studies conducted in Bangladesh [8, 9]. It has been shown that diabetes related knowledge levels are acceptable for the general public [6] and suboptimal for semi-urban participants [29]. However, a KAP survey among the general population of rural Bangladesh has found that a low levels of knowledge were associated with poor diabetes management and its risk factors [33]. Several studies have reported that knowledge about diabetes is generally poor among diabetic patients [15, 34–37]. On the contrary, a study from Malaysia identified good knowledge, attitude and practice scores among T2DMs [22]. It is therefore evident that the difference in the knowledge levels among all participants is directly related to the level of literacy, level of training received and availability of information on diabetes [38]. One possible reason for failure to answer questions correctly may reflect not just poor knowledge but also substantial misconceptions surrounding issues like incurability of diabetes, the use of sweets by diabetics and the high cost of medicine. Nonetheless, patients without diabetes were generally able to identify the symptoms and complications of diabetes, although they were not well versed in the risk factors that may lead to diabetes.

In should be noted that we observed a gender gap in knowledge, attitude and practice regarding diabetes. There are inequalities in education, health services and in all other spheres of life, with the literacy level of females lower than that of their male counterparts. Males showed significantly higher levels of knowledge, but females showed better attitudes and practice compared to males. The findings show similarities with those from studies of various countries, including Bangladesh [8, 15, 33] where a wide gender gap was evident in knowledge, attitude and practice regarding diabetes [39]. The present findings have some differences with few other studies [40–42]. However, most of these studies dealt with study participants who were already diagnosed with diabetes and attending hospitals or diabetes care centres. Thus the responses may be biased when compared with our findings from nonDM populations. It is clear that if prevention is to be effective diabetes education needs also to reach them who are still not enrolled in or engaged with a health care centre.

It is important to note that subjects from obese and overweight groups had a significantly better knowledge and practice scores (*p* < 0.000) compared to the normal and underweight groups. The attitude score, however, did not differ between the corresponding groups (*p* < 0.05). The present study shows a significant positive correlation between knowledge, attitude and practice. Better knowledge is associated with a better attitude (*r* = 0.038, *p* = 0.000) and practice (*r* = 0.314, *p* = 0.000) and better attitude is associated with better practice (*r* = 0.129, *p* = 0.000). This means that ‘the higher their knowledge the better their attitude’ is towards diabetes. These findings agree with the findings of other studies [43, 44]. The differences in positive attitudes towards the treatment of DM may be largely explained by socio-demographic status.

With regard to the risk factors of explaining the variance between poor knowledge, attitudes and practice, a study reported gender, age and socio economic status (SES) effects on knowledge [15]. In the present study higher total knowledge scores has been found for males (males 42.64 ± 15.77 vs females 40.19 ± 15.50, *p* < 0.001) and among people with a post-graduate education (postgraduate education 50.28 ± 15.83 vs upto 8th grade education 37.57 ± 14.04, *p* < 0.001). A significant difference has also been found in attitudes towards the ability to self-managed between gender, age groups, BMI and income levels. Scores for both knowledge and attitude towards treatment for diabetes in our population were in line with the UAE study [15]. Of all significant correlates of knowledge and practice, education is the only modifiable risk factor.

The correlation factors for knowledge (age, sex, area, education and income), as shown in Table 3, seems to be not a strong determinant as only 19.9% (Ra^2^) of the variation in knowledge has been explained by these variables. The situation became progressively worse for attitude and practice. It is evident that there is a need for consideration of other factors. Some of the hypothesized factors might be: disease severity, follow-up regularity and duration of disease.

Regarding self-care practices, it should be a matter of concern that only 16% of diabetic respondents have good scores in this area, meaning that only a minority group do exercise, monitor their blood glucose and follow the dietary advice regularly. There are many cases of misinformation regarding nutritional advice for people with diabetes in Bangladesh, such as ‘reduction of sugar and carbohydrates as the only means to control diabetes’. The results indicate that better practice gradually increases with better educational levels and better income in both nonDM and T2DM respondents. The majority of respondents (70%) are aware of behavioural practices, including increasing exercise, intake of more vegetables, whole grains, specific fruits and limited legumes and reducing the intake of highly refined food, and stopping smoking. The knowledge scores regarding visit to healthcare centres is found to be satisfactory. This is likely to be a result of the family history of diabetes and the counselling they receive at each visit from diabetes.

### Study strengths and limitations

This study included urban, semi-urban and rural areas and it also explored KAP in a fairly large number of DM subjects. It should be mentioned that the hospitals where the present study was conducted attracts patients with different demographic and socioeconomic backgrounds. However, as it was outpatient hospital based, the results may not be truly representative of all nonDM and all diagnosed T2DM patients in Bangladesh. In addition, due to the use of the purposive sampling technique, there may be a bias in the conclusion as neither the health centers nor the subjects were randomised. Accordingly, the reliability and generalizability of the conclusion is limited. The study was conducted in the healthcare centres run by the hospitals, where DM related education may be more readily accessible to patients. It raises concerns that DM patients and their relatives attending primary health care centres in the region with less access to diabetes education may have even poorer diabetes awareness and practices. Therefore, various issues need to be addressed in order to close the gaps between KAP. Although education is considered as an integral part of diabetes management, it remains low in the practical priorities of clinicians. The results of this study encourage a positive outlook: all that is required is trained diabetes educator in diabetes management to counsel patients during their every visit. As a result it is expected that counseling may have an impact in improving the perception about disease, diet, and lifestyle changes and thereby on glycemic control to prevent the complications of diabetes. This is also noticeable that participants came from a variety of areas, from rural to urban, but were under a single umbrella of parallel health care support system of BADAS. However all participants had similar access to information and to readily accessible education. Therefore considering the average illiteracy rate (40.18%) in Bangladesh [45], recent study findings regarding KAPs among rural populations [33], and the demographic spread of the study participants from rural to urban, the findings of this study may be considered to represent those of the general population.

## Conclusions

The study shows average level of diabetes awareness and good level of positive attitudes towards the importance of diabetes care. At the same time it has found moderate levels of diabetes practice in Bangladesh. There is a need to carry out large-scale awareness programs, after identifying the appropriate means to spread the message to the general population. There is also a need to develop of innovative tools and educational models that improve patient compliance and practices. Education and counseling on all aspects of diabetes is needed. Planning for group as well as individual education programs will deliver preventative and management techniques for DM. There is room for practice to be improved by the provision of adequate information, increasing the availability of educational materials and proper guidance towards diabetes management. The study reinforces the view that the main approach to managing this problem is to improve all stakeholders’ understanding, compliance and management of the disease by means of suitable health provisions and widespread educational campaigns.
